# Understanding the Mechanism of the Intramolecular Stetter Reaction. A DFT Study

**DOI:** 10.3390/molecules17021335

**Published:** 2012-02-02

**Authors:** Luis R. Domingo, Ramón J. Zaragozá, Jose A. Saéz, Manuel Arnó

**Affiliations:** Departamento de Química Orgánica, Universidad de Valencia, Dr. Moliner 50, E-46100 Burjassot, Valencia, Spain

**Keywords:** organocatalysis, *N*-heterocyclic carbenes, *umpolung* reactivity, intramolecular Stetter reaction, intramolecular Michael addition, mechanisms, DFT calculations

## Abstract

The mechanism of the *N*-heterocyclic carbene (NHC)-catalyzed intramolecular Stetter reaction of salicylaldehyde **1** to yield chromanone **3** has been theoretically studied at the B3LYP/6-31G** level. This NHC-catalyzed reaction takes place through six elementary steps, which involve: (i) formation of the Breslow intermediate **IN2**; (ii) an intramolecular Michael-Type addition in **IN2** to form the new C-C σ bond; and (iii) extrusion of the NHC catalyst from the Michael adduct to yield chromanone **3**. Analysis of the relative free energies in toluene indicates that while formation of Breslow intermediate **IN2** involves the rate-determining step of the catalytic process, the intramolecular Michael-type addition is the stereoselectivity determining step responsible for the configuration of the stereogenic carbon α to the carbonyl of chromanone **3**. An ELF analysis at TSs and intermediates involved in the Michael-type addition allows for the characterization of the electronic changes along the C-C bond-formation.

## 1. Introduction

The *umpolung* reactivity of aldehydes promoted by *N*-heterocyclic carbenes (NHCs) constitutes an important class of organocatalysis and has found a broad range of applications in synthetic organic chemistry [1,2,3,4,5,6]. The corresponding acyl anions or equivalent homoenolate intermediates are able to attack nucleophilically various electrophiles, such as aldehydes [7,8,9], ketones [10,11,12,13,14,15,16,17,18], imines [19,20,21,22], and even activated polarized C=C double bonds [23,24].

**Scheme 1:**
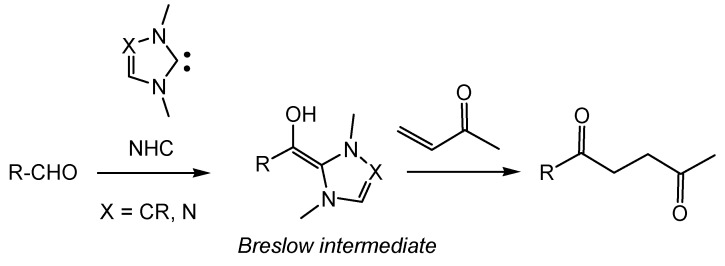
NHC-catalyzed intermolecular Stetter reaction.

The latter class of reaction is especially interesting, since it permits the *umpolung* C–C coupling between aldehydes and an appropriate Michael acceptor. In the Stetter reaction, originally conceived in the 1970’s, a homoenolate or Breslow intermediate, which inverts the normal reactivity mode of an aldehyde, provokes a Michael-type addition to electrophilically activated C=C double bonds (see Scheme 1) [25,26,27].

**Scheme 2:**
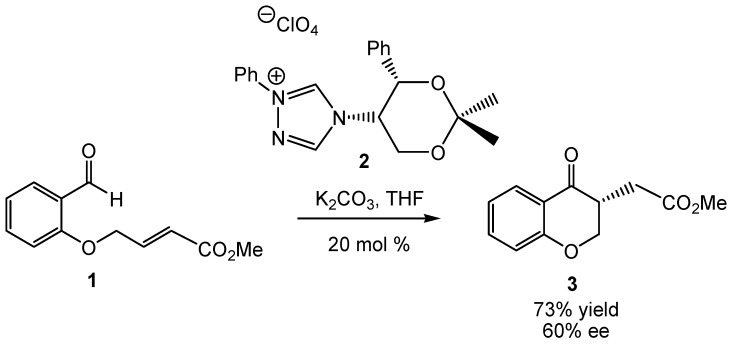
NHC-catalyzed intramolecular Stetter reaction of salicylaldehyde **1**.

Enders and co-workers were the first to report an asymmetric intramolecular Stetter reaction in 1996 [28]. Using chiral triazolium salt **2**, chromanone **3** was obtained in moderate yield (73%) and enantioselectivity (60% *ee*) (see Scheme 2). Despite the moderate selectivity, the implementation of chiral triazolinylidene carbenes in the asymmetric Stetter reaction laid the foundation for future works. Hence, Rovis performed an extensive study to improve yield and enantioselectivity in asymmetric intramolecular Stetter reactions using more efficient chiral triazolium salts, different salicylaldehyde derivatives and various reaction conditions [29,30,31,32,33].

**Scheme 3:**
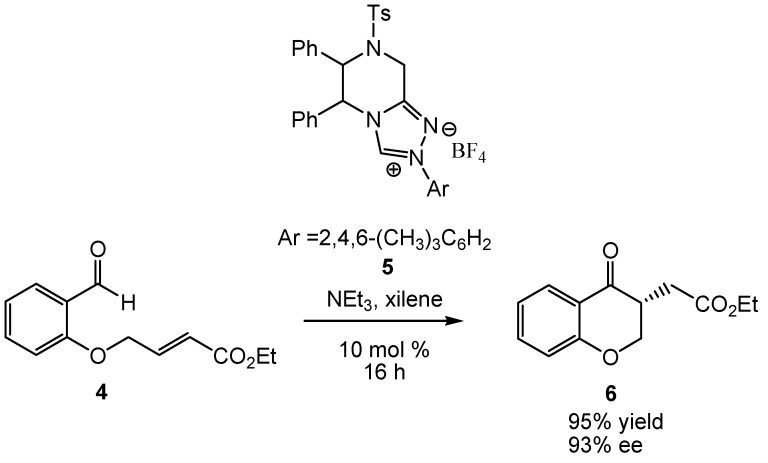
NHC-catalyzed intramolecular Stetter reaction of salicylaldehyde **4**.

Very recently, You *et al.* [34] reported the synthesis of some (1*R*,2*R*)-DPEN-derived triazolium salts such as **5**, which favor the intramolecular Stetter reaction with excellent yields and stereoselectivities of up to 97% *ee* (see Scheme 3). Several conventional bases were used, NEt_3_ being optimal in terms of both yield and *ee* of the product [34]. Solvents such as xylene, CH_2_Cl_2_, THF, Et_2_O and toluene were tested, but xylene led to an optimal combination of 95% yield and 93% *ee* [34].

**Scheme 4:**
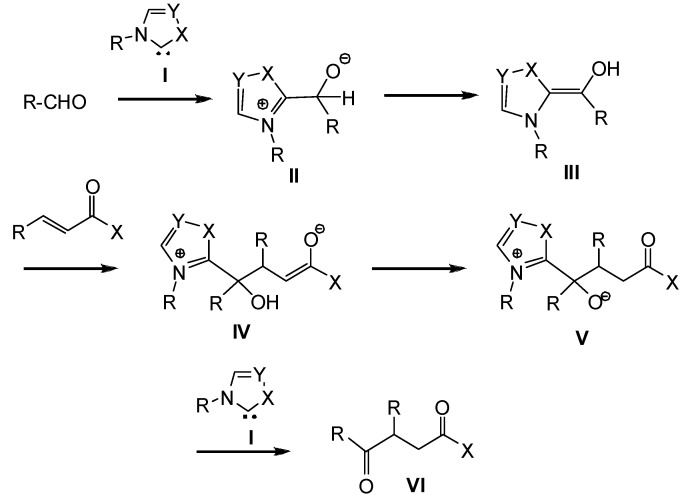
Proposed catalytic cycle for the intermolecular Stetter reaction.

Substitution effects on the aromatic ring of the salicylaldehyde structure and the nature of the tether have been analyzed. Thus, substrates bearing electron-donating groups in the salicylaldehyde structure are well tolerated and lead to their corresponding chromanone derivatives in good to excellent yields (87%–98%) and generally high *ees* (88%–97%). However, the presence of electron-withdrawing groups provide the corresponding chromanone derivatives with good yields but with relatively low *ees* [34]. Moreover, the use of oxygen, sulfur, nitrogen and carbon linkers, and the incorporation of various tethered Michael acceptors including amides, esters, thioesters, ketones, aldehydes and nitriles do not modify substantially yields and/or enantioselectivities [32]. The proposed catalytic cycle for the intermolecular Stetter reaction is shown in Scheme 4. 

NHC **I**, formed *in situ* by base deprotonation of the corresponding azolium salt, adds to the aldehyde to form the zwitterionic intermediate **II**. A proton transfer process generates Breslow intermediate **III**. Subsequent intermolecular addition to the Michael acceptor moiety forms the new C-C σ bond to generate intermediate **IV**, which by a hydrogen transfer process provides **V**. Finally, extrusion of the NHC catalyst **I** from the tetrahedral intermediate **V** affords the Stetter product **VI**.

Very recently, Rovis reported an experimental mechanistic investigation of the enantioselective intramolecular Stetter reaction, finding that the proton transfer from tetrahedral intermediate **II**, formed upon nucleophilic attack of the carbene on the aldehyde to yield Breslow intermediate **III**, is the first irreversible step [35].

**Scheme 5:**
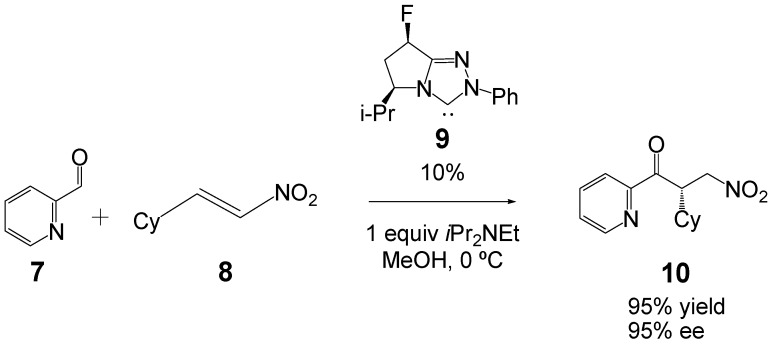
NHC-catalyzed intermolecular Stetter reaction of nitroalkene **8**.

The intermolecular Stetter reaction has been theoretically studied [36,37,38]. Very recently, Houk *et al.* studied the effect of catalyst fluorination in asymmetric Stetter reactions (see Scheme 5) [38]. They found that the more favorable transition state strucutures (TSs) associated with the Michael-type addition of the corresponding Breslow intermediate to the β-conjugated position of nitroalkene **8** exhibited a stabilizing interaction between the Breslow hydroxyl hydrogen and the carbon in the α-position to the nitro group of **8**. Note that the hydrogen-bond (HB) does not only catalyze the addition by an increase of the electrophilic character of nitroalkene **8**, but also favors the stereoselective addition in a single step.

**Scheme 6:**
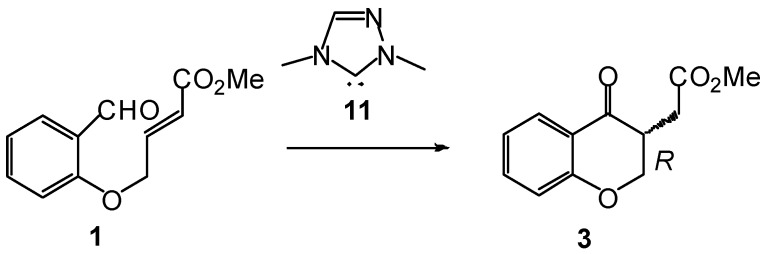
Reaction model of the NHC-catalyzed intramolecular Stetter reaction of salicylaldehyde **1**.

Our interest in organocatalysis, more specifically in the participation of NHCs as catalysts in the *umpolung* reactivity of aldehydes, prompted us to perform some theoretical studies on the molecular mechanisms of these reactions [39,40,41]. In the present manuscript, we report a DFT study of the molecular mechanism of the intramolecular Stetter reaction of salicylaldehyde **1** to yield (*R*)-chromanone **3** (see Scheme 6). An ELF analysis of the electron reorganization along the intramolecular Michael-type addition is performed in order to understand the C-C bond-formation step.

## 2. Computational Methods

DFT calculations were carried out using B3LYP [42,43] exchange-correlation functionals, together with the standard 6-31G** basis set [44]. Optimizations were performed using the Berny analytical gradient optimization method [45,46]. The stationary points were characterized by frequency calculations in order to verify that TSs had one and only one imaginary frequency. The intrinsic reaction coordinate (IRC) [47] paths were traced in order to check the energy profiles connecting each TS to the two associated minima of the proposed mechanism using the second order González-Schlegel integration method [48,49]. Values of free energies in toluene were calculated with the standard statistical thermodynamics at 298.15 K and 1 atm over the optimized gas phase structures [44]. Toluene solvent effects were considered by single point energy calculations using a self-consistent reaction field (SCRF) [50,51] based on the polarizable continuum model (PCM) of Tomasi’s group [52,53,54]. The UFF radii model was used to generate the molecular cavity in PCM calculations. The electronic structures of stationary points were analyzed by the natural bond orbital (NBO) method [55,56] and by the topological analysis of the ELF, *η*(**r**) [57,58,59]. The ELF study was performed with the TopMod program [60] using the corresponding monodeterminantal wavefunctions of the selected structures of the IRC. All calculations were carried out with the Gaussian 03 suite of programs [61].

The global electrophilicity index [62], ω, is given by the following simple expression, ω = (μ^2^/2η), in terms of the electronic chemical potential μ and the chemical hardness η. Both quantities may be approached in terms of the one electron energies of the frontier molecular orbital HOMO and LUMO, ε_H_ and ε_L_, as μ ≈ (ε_H_ + ε_L_)/2 and η ≈ (ε_L_ − ε_H_), respectively [63]. Recently, we have introduced an empirical (relative) nucleophilicity index, N [64,65], based on the HOMO energies obtained within the Kohn-Sham scheme [66], and defined as N = E_HOMO(Nu)_ − E_HOMO(TCE)_. The nucleophilicity is referred to tetracyanoethylene (TCE), because it presents the lowest HOMO energy in a large series of investigated molecules. This choice allows for the convenient handling of a nucleophilicity scale of positive values [64]. Local electrophilicity [67] and nucleophilicity [68] indices, ω_k_ and N_k_, were evaluated using the following expressions: *ω_k_* = *ωf_k_*^+^ and *N_k_* = *Nf_k_*^−^ where *f_k_*^+^ and *f_k_*^−^ are the Fukui functions [69] for nucleophilic and electrophilic attacks, respectively [70].

Very recently, we proposed a local reactivity difference index R_k_ [71] able to predict the local electrophilic and/or nucleophilic activation within an organic molecule, which is defined as [71]:

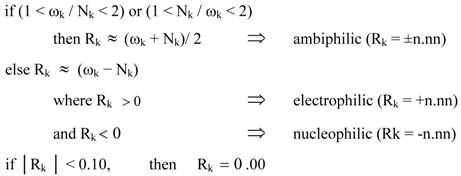

In the R_k_ index, the sign (+, −, ±) indicates the electrophilic or/and nucleophilic character of the center k, while the magnitude n.nn provides a measure of the local activation [71].

## 3. Results and Discussion

The study of the NHC-catalyzed intramolecular Stetter reaction of salicylaldehyde **1** to yield (*R*)-chromanone **3** has been divided into three parts: (*i*) energy and geometrical analysis of stationary points involved in the intramolecular Stetter reaction; (*ii*) analysis of the reaction based on DFT reactivity indices; and (*iii*) ELF topology analysis of the bonding along the intramolecular Michael-type addition of Breslow intermediate **IN2**.

### 3.1. Energy and Geometrical Analysis of Stationary Points Involved in the Intramolecular Stetter Reaction of Salicylaldehyde ***1***

**Scheme 7:**
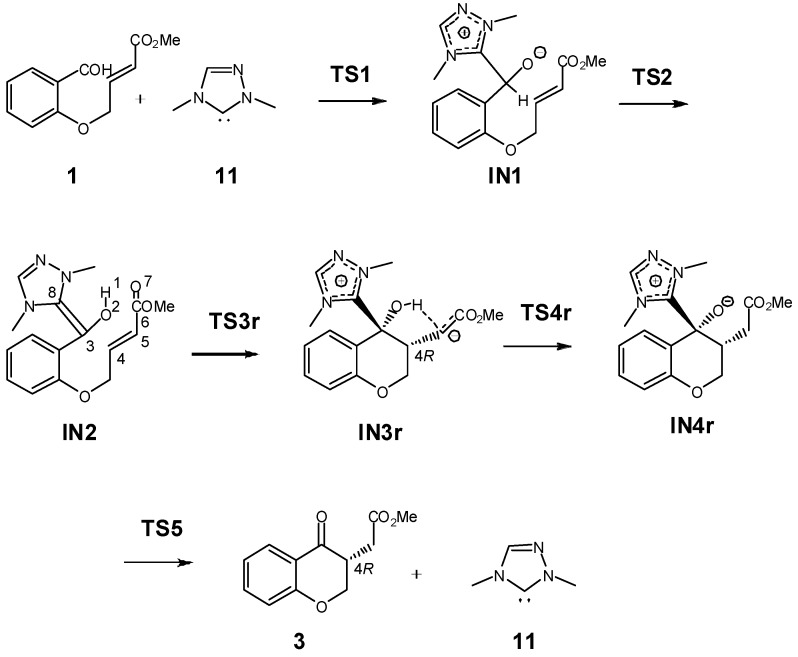
Reaction mechanism of the intramolecular Stetter reaction.

**Figure 1:**
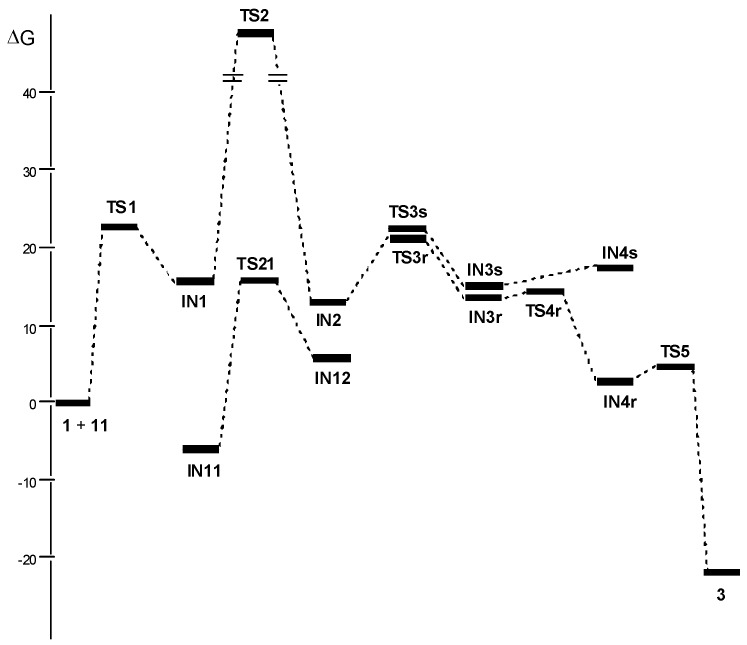
Free energy profile, in kcal/mol, of the stationary points involved in the NHC-catalyzed intramolecular Stetter reaction of salicylaldehyde **1**. Relative free energies of **TS21** and **IN12** are respect **IN11**.

The NHC-catalyzed intramolecular Stetter reaction of salicylaldehyde **1** to yield chromanone **3** comprises several elementary steps (see Scheme 7). The first one is the nucleophilic attack of NHC **11** on the carbonyl C3 carbon of salicylaldehyde **1** to yield the zwitterionic intermediate **IN1**, which by a proton transfer affords Breslow intermediate **IN2**. The subsequent intramolecular Michael-type addition to the conjugated C4 carbon of intermediate **IN2** yields ol-enolate **IN3r**, which experiences a hydrogen transfer to afford alcohoxy intermediate **IN4r**. Finally, extrusion of NHC catalyst **11** from **IN4r** provides chromanone **3**. The relative enthalpies and free energies in toluene associated with this NHC-catalyzed reaction are given in Table 1, while a schematic representation of the energy proﬁle is shown in Figure 1. The energy discussion will be made on the basis of solvent free energies in toluene.

**Table 1:** Total (H and G, in au) and relative (ΔH and ΔG, in kcal/mol) enthalpies and free energies, computed at 25 °C and 1 atm in toluene, of the stationary points involved in the intramolecular Stetter reaction of salicylaldehyde **1**.

	H	ΔH	G	ΔG
**1**	−765.154909		−765.216688	
**11**	−320.722503		−320.761367	
**TS1**	−1085.864102	8.4	−1085.943426	21.7
**IN1**	−1085.875090	1.5	−1085.953173	15.6
**TS2**	−1085.807807	43.7	−1085.885314	58.2
**IN2**	−1085.879507	−1.3	−1085.957544	12.9
**TS3r**	−1085.873415	2.5	−1085.944874	20.8
**IN3r**	−1085.884019	−4.1	−1085.955978	13.9
**TS3s**	−1085.870106	4.6	−085.941886	22.7
**IN3s**	−1085.884133	−4.2	−1085.954410	14.8
**TS4r**	−1085.885839	−5.3	−1085.956900	13.3
**IN4r**	−1085.900792	−14.7	−1085.973665	2.8
**IN4s**	−1085.878805	−0.9	−1085.949454	17.9
**TS5**	−1085.896768	−12.1	−1085.970371	4.8
**3**	−765.194373	−24.8	−765.251292	−21.7

**Scheme 8:**
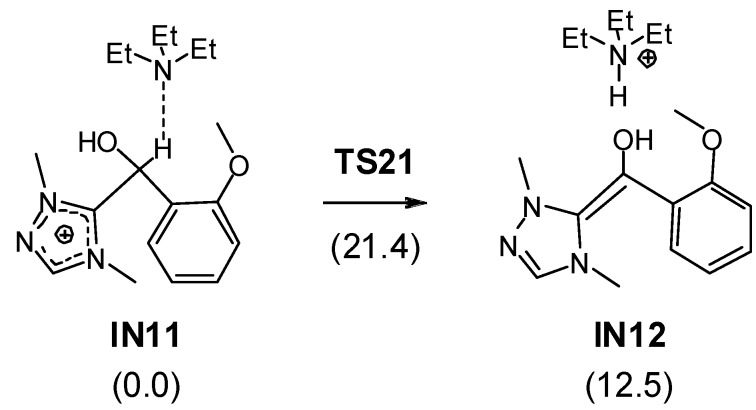
Intermolecular hydrogen abstraction associated with the formation of Breslow intermediates.

**TS1,** associated with the nucleophilic attack of the C8 carbon of NHC **11** on the C3 carbon of salicylaldehyde **1**, presents a free activation energy of 21.7 kcal/mol; formation of the zwitterionic intermediate **IN1** is endergonic by 15.6 kcal/mol. The subsequent proton transfer from the aldehyde C3 carbon to the O2 oxygen at the zwitterionic intermediate **IN1** affords Breslow intermediate **IN2**. Recently, we have shown that direct proton transfer with formation of Breslow intermediates such as **IN2** have a very high free activation energy as a consequence of the strain associated with the three-membered TSs [39,41]; accordingly, the free activation energy associated with the proton transfer via **TS2** is very high, 42.6 kcal/mol. Several acid/base species can catalyzed the conversion of zwitterionic intermediate **IN1** into Breslow intermediate **IN2** [39,41], including the NEt_3_/NEt_3_H^+^ pair resulting from the deprotonation of the triazolium salts [34]. We tested this possibility in order to estimate the free activation energy associated with the proton transfer. Since the unsaturated ester appendage present in salicylaldehyde **1** does not participate in this process, we used a reduced model in which the unsaturated ester present in **IN1** was replaced by a methyl group (see Scheme 8, relative free energies in toluene are given in parentheses). The intermolecular hydrogen transfer process takes place in two steps: (i) protonation of the alcohoxy O2 oxygen by triethyl ammonium cation; and (ii) abstraction of the H1 hydrogen by triethylamine. The first step is barrierless and strongly exothermic due to the more basic character of the alcohoxy O2 oxygen than triethylamine. However, the subsequent H1 hydrogen abstraction has an appreciable activation free energy; 21.4 kcal/mol from **IN11** plus NEt_3_. Therefore, this energy barrier, which is similar to those found in formation of Breslow intermediates catalyzed by methanol [39,41], indicates that the proton transfer is the rate-determining step in formation of Breslow intermediate **IN2**. From **1** plus **11**, formation of Breslow intermediate **IN2** is endergonic by 12.9 kcal/mol.

**Scheme 9:**
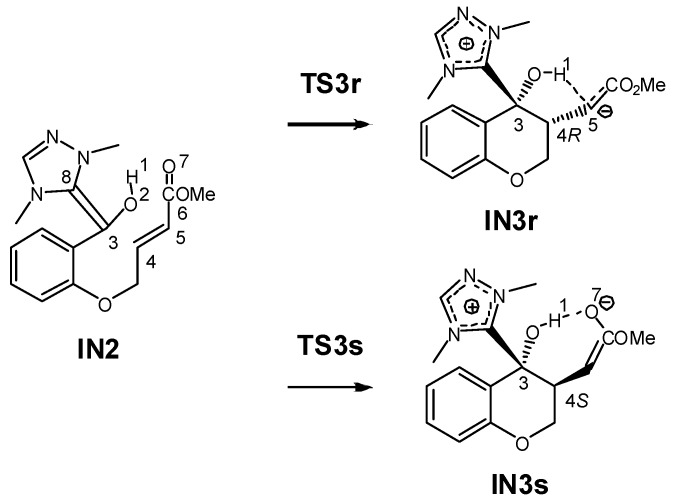
Competitive channels associated with the intramolecular Michael addition in **IN2**.

The next step is the intramolecular nucleophilic attack of the C3 carbon belonging to the Breslow framework on the conjugated C4 carbon of the unsaturated ester framework present in **IN2** to yield the ol-enolate **IN3r**. For the intramolecular Michael-type addition, which is the stereoselectivity determining step responsible for the *R* or *S* configuration at the stereogenic carbon α to the carbonyl carbon of chromanone **3**, four stereoisomeric channels related to the nucleophilic attack of the *re-* or *si-face* of the C3 carbon of the Breslow framework of **IN2** on the *re-* or *si-face* of the conjugated C4 carbon of the unsaturated ester residue are feasible. In experimental Breslow intermediates, both the nucleophilic C3 carbon and the electrophilic C4 carbon are prochiral centers. As the C8 environment at NHC **11** is symmetric, this NHC cannot induce diastereoselectivity and then, only the two stereoisomeric channels associated with the attack of the *si-face* of the C3 carbon of the Breslow framework on the *re-* or *si-face* of the conjugated C4 carbon of the unsaturated ester residue were considered (see Scheme 9). Consequently, two TSs, **TS3r** and **TS3s**, and the corresponding Michael zwitterionic intermediates **IN3r** and **IN3s**, allowing for the *R* and *S* configuration at the stereogenic carbon α to the carbonyl carbon of chromanone **3**, were studied (see Scheme 9). From Breslow intermediate **IN2**, the free activation energies associated with **TS3r** and **TS3s** are 7.9 and 9.8 kcal/mol, respectively. Formation of Michael intermediates **IN3r** and **IN3s** is endergonic by 1.0 and 1.9 kcal/mol. The free energy difference between **TS3r** and **TS3s**, 1.9 kcal/mol, may be attributed to the preferential HB formation to the C5 carbon of the enolate at **TS3r** rather than to the O7 oxygen atom at **TS3s**. Further study using a more complex NHC model would be needed to disclose the stereoselectivity origin. **IN3r**, with negative free activation energy, −0.5 kcal/mol, experiences a fast hydrogen transfer process via **TS4r** to yield the Michael adduct **IN4r**. From Breslow intermediate **IN2**, formation of **IN4r** is strongly exergonic, −10.1 kcal/mol. This behavior makes the C3-C4 bond-formation step irreversible. All attempts to obtain enolester **IN4s** as a stationary point were unsuccessful as it reverts to **IN3s**. Restricted optimization of **IN4s** fixing the O7-H1 distance yields a species which is 3.1 kcal above **IN3s**. Finally, intermediate **IN4r** experiences an easy extrusion of NHC catalyst **11** to yield chromanone **3**, through a low free activation energy process, 2.0 kcal/mol, via **TS5**. From the separated reagents, **1** plus **11**, formation of chromanone **3** is strongly exergonic, −21.7 kcal/mol.

Analysis of the free energy profile given in Figure 1 shows that while formation of Breslow intermediate **IN2** is endergonic, formation of Michael-type adduct **IN4r**, and chromanone **3**, from **IN2** and **IN4r**, are exergonic processes. As the proton transfer in **IN1** is the most unfavorable step, formation of Breslow intermediate **IN2** is the rate-determining step. This behavior is in agreement with Rovis’s recent proposal that the proton transfer from tetrahedral intermediate **IN1** is the first irreversible step [35]. On the other hand, once **IN2** is formed, the reaction progresses irreversibly. The lower free energy found at **TS4r** than at **TS3r** together with the irreversible character of the intramolecular Michael-type addition prompts the nucleophilic attack of the C3 carbon on the electrophilic C4 carbon of **IN2** via **TS3r**, the stereoselectivity determining step responsible for the configuration of stereogenic carbon α to the carbonyl carbon of chromanone **3**.

**Figure 2:**
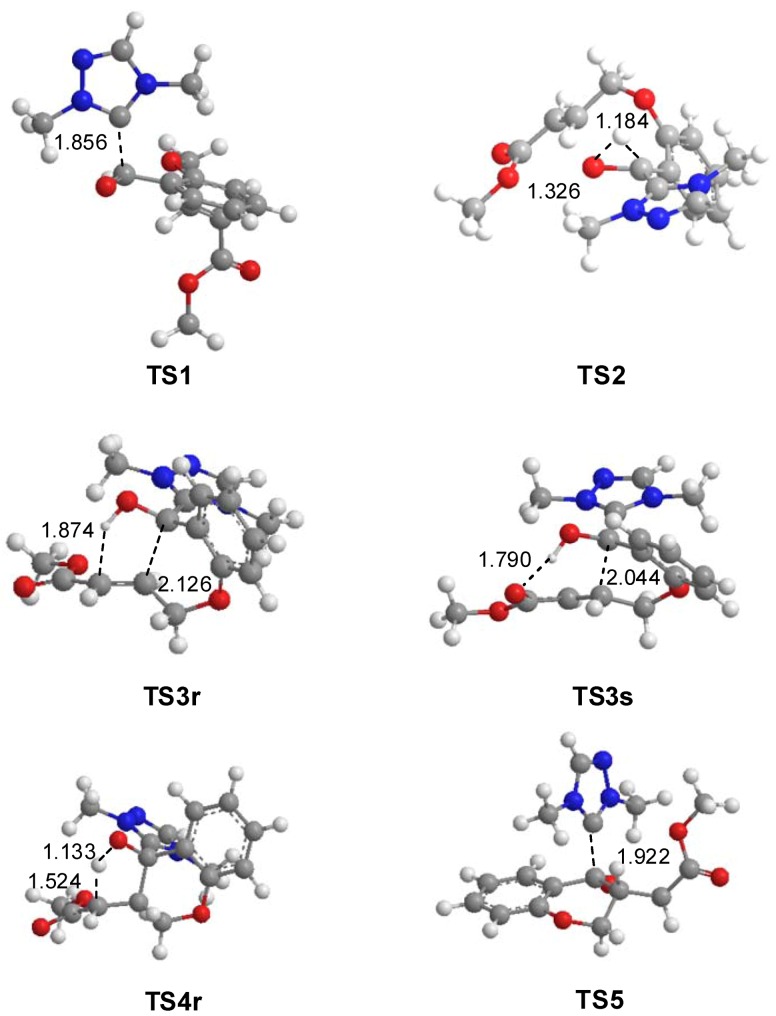
Transition structures associated with the NHC-catalyzed intramolecular Stetter reaction of salicylaldehyde **1**. The distances are given in Å.

The gas-phase geometries of the TSs involved in the NHC-catalyzed intramolecular Stetter reaction are given in Figure 2. At **TS1**, associated with the nucleophilic attack of the C8 carbon of NHC catalyst **11** on the carbonyl C3 carbon of salicylaldehyde **1**, the length of the C3–C8 forming bond is 1.856 Å. At **TS2**, associated with the intramolecular proton transfer process, the lengths of the C3-H1 breaking and O2-H1 forming-bond are 1.184 Å and 1.326 Å, respectively. At the stereoisomeric TSs associated with the intramolecular Michael-type addition of the Breslow framework to the β-conjugated position of the unsaturated ester framework of **IN2**, the lengths of the C3–C4 forming bonds are 2.126 Å (**TS3r**) and 2.044 Å (**TS3s**). While at **TS3r** the distance between the hydroxyl H1 hydrogen and the C5 carbon of the unsaturated ester is 1.874 Å, at **TS3s** the distance between the hydroxyl H1 hydrogen and the carboxyl O7 oxygen is 1.790 Å. These short distances point to strong HB interactions as a consequence of the negative charge that is being transferred towards the unsaturated ester framework. At **TS4r**, associated with the intramolecular hydrogen transfer process, the lengths of the O2-H1 breaking and C5–H1 forming bonds are 1.133 Å and 1.524 Å, respectively. These values indicate that the TS has an early character. At **TS5** associated with the extrusion of NHC catalyst **11**, the length of the C3–C8 breaking bond is 1.922 Å. Finally, at **TS21** associated with the hydrogen elimination in intermediate **IN11**, the lengths of the C3-H1 breaking- and N1-H forming-bonds are 1.544 Å and 1.255 Å, respectively.

### 3.2. Analysis of the Reaction Based on DFT Reactivity Indices

Analysis of the reactivity indices defined within the conceptual DFT allows for the understanding of polar reactions. The static global properties of the species involved in the nucleophilic/electrophilic interactions along the NHC-catalyzed intramolecular Stetter reaction of salicylaldehyde **1**, namely electronic chemical potential (μ), chemical hardness (η), global electrophilicity (ω), and global nucleophilicity (N), are given in Table 2.

**Table 2:** Electronic chemical potential (μ, in a.u.), chemical hardness (η, in a.u.), global electrophilicity (ω, in eV), and global nucleophilicity (N, in eV) of salicylaldehyde **1**, NHC **11** and Breslow intermediate **IN2**.

	μ	η	ω	N
Benzaldehyde	−0.1590	0.1923	1.79	2.18
**1**	−0.1503	0.1752	1.75	2.65
Methyl acrylate	−0.1586	0.2267	1.51	1.72
**IN2**	−0.0975	0.0974	1.33	5.14
**11**	−0.0964	0.2334	0.54	3.32

Salicylaldehyde **1** has an electrophilicity power of 1.75 eV, which is closer to that of benzaldehyde, ω = 1.79 eV, therefore, both are classified as strong electrophiles within the electrophilicity scale [72]. Salicylaldehyde **1** has a nucleophilicity index N of 2.67 eV, being classified as a moderate nucleophile [73]. On the other hand, NHC **11** has a low electrophilicity index, ω = 0.54 eV, being classified as a marginal electrophile, while it is a strong nucleophile, N = 3.32 eV [39]. Consequently, it is expected that along the nucleophilic attack of NHC **11** on salicylaldehyde **1** there will be a strong nucleophile/electrophile interaction, favoring the formation of tetrahedral intermediate **IN1**.

Recent studies devoted to intramolecular Diels-Alder reactions have shown that the analysis of the global electrophilicity and nucleophilicity indices at the ground state of reagents is able to predict the polar character of these intramolecular reactions [74,75]. The electrophilicity of Breslow intermediate **IN2**, ω = 1.33 eV, allows for its classification as a moderate electrophile. Note that the electrophilicity of **IN2** is closer to that of methyl acrylate ω = 1.51 eV; On the other hand, **IN2** has a high nucleophilicity index of 5.14 eV, thus being classified as a strong nucleophile. Consequently, it is expected that the intramolecular Michael-type addition in **IN2** will take place through a strong nucleophile/electrophile electronic interaction, which will favor a polar process. Note that the electrophilicity of intermediate **IN2** is increased through the formation of intramolecular HBs of the H1 hydrogen with the C5 carbon or O7 oxygen along the intramolecular Michael-type addition (see Scheme 8).

**Scheme 10:**
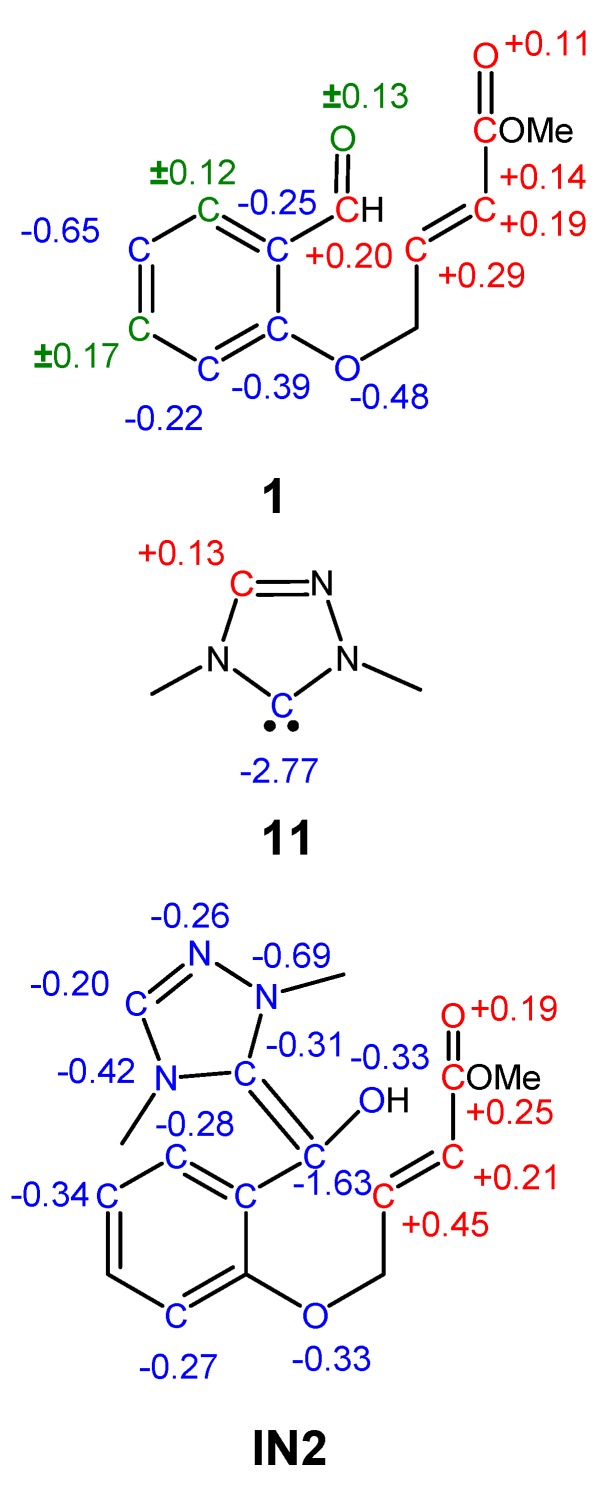
RMMRs of salicylaldehyde **1**, NHC catalyst **11** and Breslow intermediate **IN2**.

Recently, we have proposed a local reactivity difference index R_k_ able to predict the local electrophilic and/or nucleophilic activation within an organic molecule [71].Together with the electrophilic and/or nucleophilic behavior of the k center, denominated by its sign, the magnitude of the R_k_ index accounts for the extent of the electronic activation. The representation of the significant R_k_ indices, |R_k_| > 0.10 eV, in a molecule constitutes the R_k_ molecular map of reactivity (RMMR) [71]. The RMMRs of salicylaldehyde **1**, NHC catalyst **11** and Breslow intermediate **IN2** are given in Scheme 10.

Salicylaldehyde **1** has the most electrophilically activated sites at the carbonyl C3 carbon, R_C3_ = +0.20 eV, and at the conjugated C4 carbon, R_C4_ = +0.29 eV. Althrough the C4 carbon is more electrophilically activated than the C3 one, the reversible nucleophilic attack of **11** at the C4 carbon and the irreversible nucleophilic attack at C3 can shift the reaction towards **IN2**.

The C8 carbon of NHC catalyst **11** is the only nucleophilically activated center of this molecule, R_C8_ = −2.77 eV, therefore concentrating most of the nucleophilicity of the NHC, N = 3.32 eV. This behavior is due to the fact that most of the electron-density associated with the HOMO of NHC **11** is located at the sp^2^ hybridized C8 carbon.

Breslow intermediate **IN2** presents nucleophilic activation at the atoms belonging to NHC and the aldehyde frameworks, while the unsaturated ester framework shows electrophilic activation (see Scheme 10). Whereas the C3 carbon belonging to the Breslow framework is the most nucleophilic center of **IN2**, R_C3_ = −1.63 eV, the conjugated C4 carbon is the most electrophilically activated center, R_C4_ = +0.45 eV. Consequently, the most favorable nucleophilic/electrophilic interaction along the intramolecular process will take place between the C3 and C4 carbons, allowing for the C3-C4 bond-formation.

### 3.3. ELF Bonding Analysis along the Intramolecular Michael Addition in Breslow Intermediate ***IN2***

Recent theoretical studies have shown that the topological analysis of the ELF along the reaction path associated with an organic reaction is a valuable tool for understanding the bonding changes along the reaction path, and therefore, to characterize the molecular mechanism [76,77,78,79,80,81,82].

**Figure 3:**
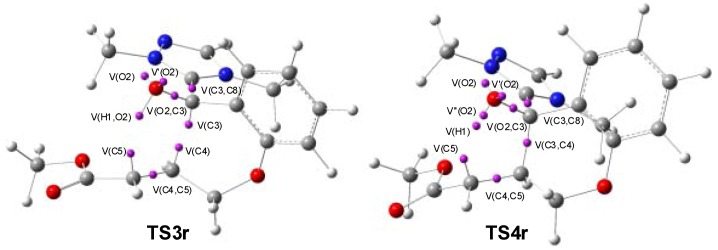
Most relevant ELF attractors at **TS3r** and **TS4r** associated with the intramolecular Michael addition in Breslow intermediate **IN2**.

Consequently, a topology analysis of the ELF of the stationary points involved in the intramolecular Michael-type addition in Breslow intermediate **IN2** was carried out in order to characterize the bond formation. The *N* populations of the more relevant ELF valence basins of the stationary points involved in the intramolecular Michael-type addition in Breslow intermediate **IN2** are listed in Table 3, while the positions of the more relevant attractors for **TS2r** and **TS3r** are shown in Figure 3.

**Table 3:** Valence basin populations *N* of the most relevant valence basins calculated from the ELF of the intramolecular Michael addition in Breslow intermediate **IN2**.

	IN2	TS3r	IN3r	TS4r	IN4r
V(C3,C8)	2.01	2.85	2.45	2.44	2.44
V’(C3,C8)	2.20				
V(O2)	2.39	2.44	2.34	2.44	2.17
V’(O2)	2.43	2.47	2.52	2.57	3.87
V’’(O2)				0.94	
V(O2,C3)	1.24	1.35	1.36	1.35	1.58
V(C4,C5)	1.76	2.79	2.02	1.99	1.85
V’(C4,C5)	1.72				
V(C3,C4)		1.04	1.85	1.86	1.96
V(C4)		0.34			
V(C5)		0.54	1.17	1.26	
V(H1,O2)	1.71	1.65	1.61		
V(H1)				0.57	
V(H1,C5)					2.01

ELF analysis of Breslow intermediate **IN2** shows one disynaptic basin V(H1,O2) and another disynaptic basin V(O2,C3), each one integrating 1.71e and 1.24e, respectively, associated with the O2-H1 and O2-C3 σ bonds, and two monosynaptic basins, V(O2) and V’(O2), integrating a total of 4.82e, which are associated with the two lone pairs of the O2 oxygen. The populations of these four valence basins show a strong polarization of the electron-density of the single O2-H1 and O2-C3 σ bonds towards the electronegative O2 oxygen. Breslow intermediate **IN2** also presents two pairs of disynaptic basins, V(C3,C8) and V’(C3,C8) and V(C4,C5) and V’(C4,C5), which integrate a total of 4.21e and 3.48e, respectively, corresponding to the C3-C8 and C4-C5 double bonds present in the Lewis structure of intermediate **IN2**.

At **TS3r**, some relevant changes take place relative to the electronic structure of Breslow intermediate **IN2** (see Figure 3). The two disynaptic basins V(C4,C5) and V’(C4,C5) merge into one disynaptic basin V(C4,C5), which accounts for 2.79e. Consequently, a strong reduction of the electron-density at the C4-C5 double bond region has taken place. Concurrently, two monosynaptic basins V(C4) and V(C5), integrating 0.34e and 0.54e, respectively, emerge at the olefinic C4 and C5 carbons. Interestingly, a new disynaptic basin V(C3,C4), integrating 1.04e, associated with the formation of the new C3-C4 σ bond, appears. Consequently, the C3-C4 σ bond is already formed at **TS3r**. On going from **TS3r** to **IN4r**, this disynaptic basin is fully populated. Finally, at **TS3r**, the two disynaptic basins V(C3,C8) and V’(C3,C8), associated with the C3-C8 double bond present at intermediate **IN2**, also merge into one disynaptic basin V(C3,C8) with a population of 2.85e.

At **IN3r**, while the monosynaptic basin V(C4) has disappeared, and the population of the disynaptic basin V(C4,C5) has decreased to 2.02e, the population of the new disynaptic basin V(C3,C4) increases to 1.85e, indicating that the new C3-C4 σ bond is almost completed. On going from **IN2** to **IN3r**, the population of the disynaptic basin V(H1,O2) decreases slightly to 1.61e.

At **TS4r** while the disynaptic basin V(H1,O2) has disappeared, two new monosynaptic basins V’’(O2) and V(H1), associated with the hydrogen transfer process, with a population of 0.94e and 0.57e, respectively, are created (see Figure 3). Note that these two monosynaptic basins come from the O2-H1 breaking bond. On the other hand, the population of the monosynaptic basin V(C5) amounts to 1.26e.

Finally, at **IN4r**, the two monosynaptic basins V(H1) and V(C5) merge into the new disynaptic basin V(H1,C5) with a population of 2.01e, indicating that the H1-C5 σ bond has been completely formed.

Taking a look at the results obtained through the ELF topology analysis at the stationary points of the NHC-catalyzed intramolecular Michael-type addition, we can see that the forming and breaking bond processes take place in two differentiated steps. Along the first step, the new C3-C4 σ bond is already created at **TS3r**, while the population of the corresponding disynaptic basin V(C3,C4) is practically completed at intermediate **IN3r**. At this step, the H1-O2 σ bond remains practically unchanged. Along the second step, the H1 hydrogen is transferred from the O2 oxygen to the C5 carbon. This second step starts at **TS4r**, where the H1-O2 σ bond is broken, yielding two new monosynaptic basins V(H1) and V’’(O2), which disappear at **IN4r** with the formation of the second C5-H1 σ bond.

## 4. Conclusions

The mechanism of the NHC-catalyzed intramolecular Stetter reaction of salicylaldehyde **1** to yield chromanone **3** has been theoretically studied at the B3LYP/6-31G** computational level. This NHC-catalyzed reaction takes place through six elementary steps. The reaction begins by the nucleophilic attack of the NHC catalyst on the aldehyde carbon of salicylaldehyde **1** to yield a tetrahedral intermediate, which in a two-step proton transfer process generates Breslow intermediate **IN2**. The subsequent intramolecular addition to the Michael acceptor moiety forms the new C-C σ bond to generate intermediate **IN3r**, which by a fast hydrogen transfer process provides alcohoxy intermediate **IN4r**. Finally, extrusion of the NHC catalyst from this intermediate affords the Stetter product **3**. Analysis of the free energy profile associated with this catalytic process indicates that while formation of Breslow intermediate **IN2** involves the rate-determining step, the intramolecular Michael-type addition in Breslow intermediate **IN2** is the stereoselectivity determining step responsible for the configuration of the stereogenic carbon α to the carbonyl carbon of chromanone **3**.

Analysis of the reactivity indices defined within the conceptual DFT verifies the high reactivity of Breslow intermediate **IN2**. Finally, an ELF bonding analysis at TSs and intermediates involved in the intramolecular Michael-type addition allows for the characterization of the bond-formation. While at **TS3r** the new C3-C4 σ bond is already formed, at **TS4r** the hydroxyl H1 hydrogen is transferred to the olefinic C5 carbon.
